# Electroreception in treehoppers: How extreme morphologies can increase electrical sensitivity

**DOI:** 10.1073/pnas.2505253122

**Published:** 2025-07-21

**Authors:** Sam J. England, Ryan A. Palmer, Liam J. O’Reilly, Isaac V. Chenchiah, Daniel Robert

**Affiliations:** ^a^Faculty of Health and Life Sciences, School of Biological Sciences, University of Bristol, Bristol BS8 1TQ, United Kingdom; ^b^Department of Evolutionary Morphology, Museum für Naturkunde–Leibniz Institute for Evolution and Biodiversity Science, Berlin 10115, Germany; ^c^Faculty of Science and Engineering, School of Engineering Mathematics and Technology, University of Bristol, Bristol BS8 1TW, United Kingdom; ^d^Faculty of Science and Engineering, School of Mathematics, University of Bristol, Bristol BS8 1UG, United Kingdom

**Keywords:** electrostatics, electroreception, insects, electric fields, predator-prey interactions

## Abstract

The link between form and function of an organism’s morphology is usually apparent or intuitive. However, some clades of organisms show remarkable diversity in their form, often exhibiting extreme morphologies, but with no obvious functional explanation. Treehoppers (Membracidae) are a family of insects that exemplify this, displaying an astounding morphological diversity, resulting in a plethora of extreme forms. The function of these morphological extremities and the reasons for their evolution have thus far remained largely enigmatic. However, this mystery can be considered in light of the capacity of many animals to detect electric fields in air via electrostatic actuation of mechanosensory structures on their body. Importantly, the strength of the electric field experienced by these mechanosensory structures is expected by physics to depend on the animal’s geometry, with sharp and elongated features producing the highest electric fields. Therefore, we hypothesize that the extreme morphologies of treehoppers increase their electrical sensitivity. Here, we show that treehoppers, along with their predators and mutualists, produce electric fields and that the treehopper *Poppea capricornis* can detect electric fields, responding behaviorally. We also demonstrate that predatory wasps and mutualist bees differ significantly in their electrostatic profiles, pointing to the sophistication of electrical information potentially available to treehoppers. Biophysical, computational, and mathematical techniques are then utilized to provide evidence that the pronotum of treehoppers is the site of electroreception and that its extreme shapes may enhance its sensitivity to electricity.

## Significance

Our study reveals that the extreme morphologies seen in animals such as treehoppers may increase their sensitivity to electrical stimuli. We show that treehoppers can likely detect the electric fields of their predators and that sufficient electrostatic information exists in the ecology of treehoppers that they may even distinguish these predators from friendly bees using electrical cues alone. This introduces a level of sophistication not previously ascribed to the electrostatic sense. Furthermore, by demonstrating that the extreme morphology of treehoppers increases the strength of electric field stimuli around these animals, we suggest that the enigmatic function of their spectacular pronota is partly as an electroreceptor and that natural selection for increased electrical sensitivity may have contributed to their diverse evolution.

Electroreception is the ability of an organism to detect ecologically relevant electric fields (1). This sensory modality was first discovered in the sharks and rays (2–5) and for a long time was thought to be an ability exclusive to fauna in aquatic environments (6). This is because electroreception in water relies upon direct electrical conduction from source to receptor. In terrestrial environments, such a mechanism is challenging because the electrical conductivity of air is typically at least 11 orders of magnitude lower than that of water. However, in the last decade, evidence has emerged demonstrating that several terrestrial species are in fact capable of detecting ecologically relevant electric fields in air, via an electrostatic mechanism that does not require electrical conduction (1, 7–17).

Aerial electroreception was first demonstrated in bumblebees, showing that they can detect the electric fields around flowers and may use this as a cue to assess floral reward and inform foraging strategy (7). It has since been shown that aerial electroreception is also used by hoverflies for floral assessment (8), as well as honeybees for intraspecific communication (11), spiders for informing dispersal decisions (12, 13), and flower mites to detect approaching hummingbirds for phoresis (10). Most pertinently here, it has recently been discovered that aerial electroreception can be utilized by caterpillars for predator detection (9). In each of these terrestrial animals, the mechanism of electroreception has been identified as electrostatic deflection of mechanosensory structures, namely setae or antennae (8, 9, 11, 12, 17). This mechanism is entirely distinct from the conductive mechanisms utilized by aquatic species, and therefore very little is known about how this electromechanical sensory system may have evolved and adapted.

**Fig. 1.**
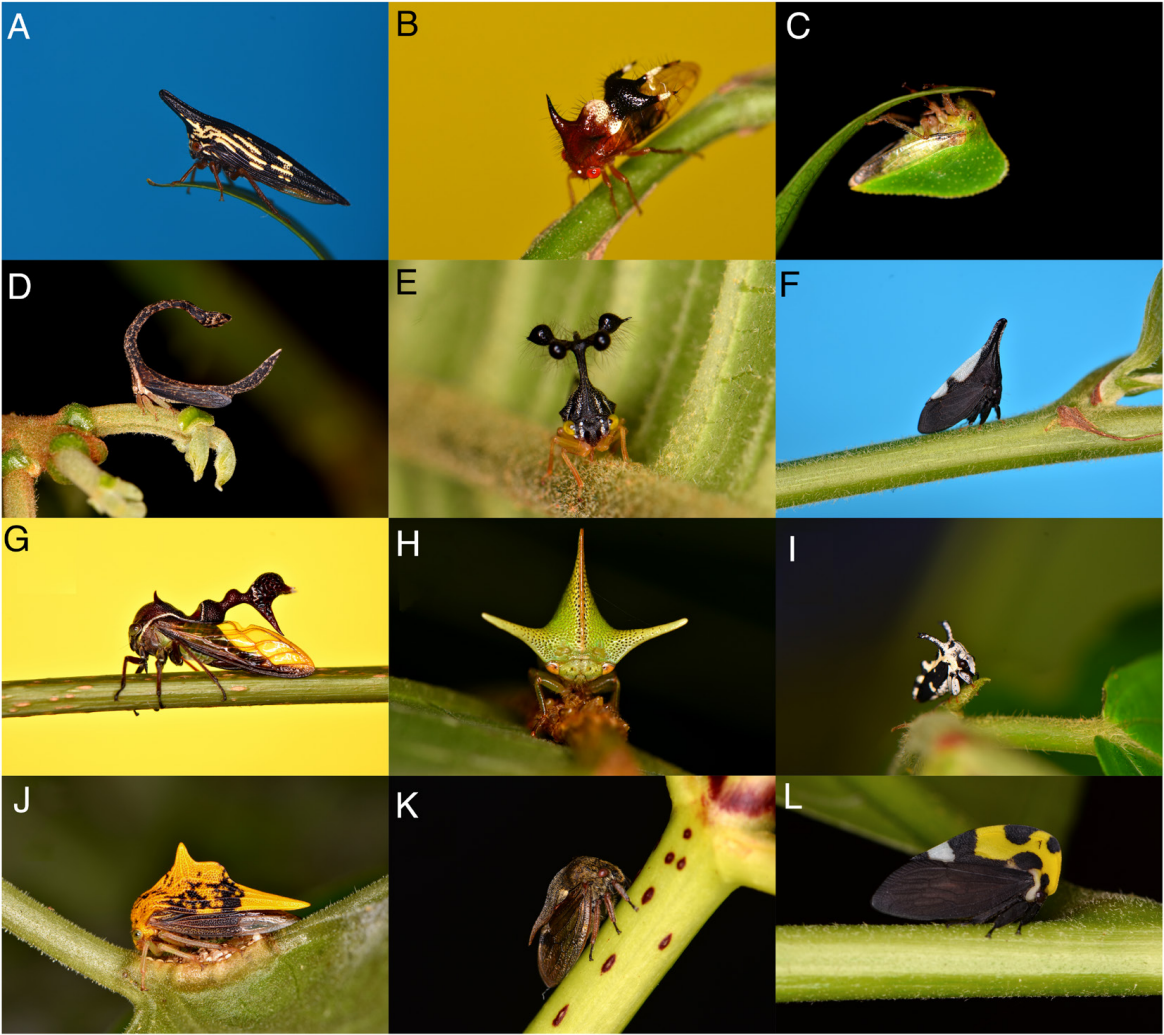
Macrophotographs of wild treehoppers exemplifying a small portion of the morphological diversity seen in the Membracidae, photographed at various locations around Costa Rica (*A*–*J* and *L*) and the United Kingdom (*K*). (*A*) *Polyglypta costata*. (*B*) *Poppea capricornis.* (*C*) *Antianthe expansa.* (*D*) *Cladonota apicalis.* (*E*) *Bocydium mae.* (*F*) *Enchophyllum* sp. (*G*) *Heteronotus trinodosus*. (*H*) *Alchisme grossa.* (*I*) *Notocera* sp. (*J*) *Ennya chrysura.* (*K*) *Centrotus cornutus*. (*L*) *Membracis mexicana*.

One key question that arises from these discoveries is whether some clades may have evolved specializations for aerial electroreception that improve the efficacy, reliability, or sensitivity of this sensory modality. To begin to test the hypothesis that adaptations have occurred for improving electroreceptive abilities in air, treehoppers (Membracidae) are highlighted here as a focal clade. Treehoppers are a speciose family of insects, with over 3,000 described species worldwide (18). The most notable feature of this family is their spectacular morphological diversity, resulting from a striking evolutionary radiation of their pronotum into a plethora of extravagant and elaborate shapes (19), examples of which can be seen in Fig. 1. The expanded pronotum of treehoppers is a highly novel trait, and its development and evolution are an ongoing source of intrigue and sometimes controversy (20–25).

Many suggestions have been made as to the function of the treehopper pronotum, including crypsis, masquerade, mimicry, visual signaling, aposematism, and physical defense (26–32). Although each of these is likely to have contributed to the morphological evolution of certain treehopper clades, there is currently little experimental evidence addressing these hypotheses (33, 34), and none of them would apply universally to the entire treehopper family. Most pertinently here, the pronotum is physiologically active and is covered with articulated setae that are innervated (35–37) and thus likely to be mechanosensory. Therefore, a universal function of the treehopper pronotum may be as a sensory system; but to what stimuli is not known. As it has previously been shown that the mechanosensory setae of other arthropods are sensitive to electric fields (8, 9, 12, 17), it is likely that the setae found on the treehopper pronotum are too. As such, we hypothesize that the treehopper pronotum is capable of functioning as an electrosensory system. More than that, we suggest that the treehopper pronotum heightens sensitivity to electrical stimuli by utilizing acute structures of high aspect ratio to concentrate the electric field on the sensory setae of the pronotum, as well as by simply increasing the available surface area for electroreceptors.

Treehoppers are heavily predated upon by both vertebrates and invertebrates (18, 29, 38–45) and also form important mutualisms with other animals, notably ants (Formicidae) and stingless bees (Meliponini), that protect them from predators in exchange for access to the honeydew that they secrete (18, 46–52). Additionally, the greatest morphological diversity of treehoppers is found in the tropics (18), where predation pressure on insects is highest (53). Together, these facts insinuate that predation could play a significant role in the evolution of the treehopper pronotum. Indeed, intraspecific variations in the size and shape of the adult treehopper pronotum are correlated with the survival of their offspring, and thus, pronotum morphology is evidently under direct natural selection (54). Therefore, we focus here on the context of predator and mutualist detection because this likely exerts the strongest selective pressure on the sensory function of the treehopper pronotum.

Herein, the hypothesis that the treehopper pronotum is utilized for aerial electroreception, and exhibits morphological advantages for this function, is explored with the use of behavioral, biophysical, morphological, computational, and mathematical techniques.

## Results

### Treehoppers, Predatory Wasps, and Mutualist Bees Carry Different Electrostatic Charges.

First, it was vital to quantify the electrostatic charges involved in the natural ecology of treehoppers. Because higher relative humidity is associated with reduced electrostatic charging in other insects (55), it was important to assess whether tropical treehoppers, as well as the predators and mutualists with which they interact, accumulate static charges despite the high humidity of their habitats. Furthermore, we wanted to establish whether the charges of these groups were significantly different from each other and thus whether treehoppers may be able to distinguish friend from foe via electroreception alone. Therefore, we measured the charges of various wild treehoppers, wasps, and bees in multiple locations around Costa Rica.

The net charges of all insects were measured using a picoammeter connected to a ring electrode system through which the insects passed, as used and described in previous studies on temperate insects (9, 56). A total of 151 individual treehoppers from 11 different evolutionary clades had their net electrostatic charge measured, namely *Poppea capricornis* (N = 18), *Polyglypta costata* (N = 9), *Notocera* sp. (N = 3), *Membracis mexicana* (N = 13), *Heteronotus trinodosus* (N = 6), *Enchophyllum* sp. (N = 3), *Cladonota apicalis* (N = 1), *Bolbonota* sp. (N = 1), *Antianthe expansa* (N = 55), *Alchisme grossa* (N = 32), and Aconophorini (N = 10). Two species of wasp (*Mischocyttarus* cf. *angulatus*, N = 8, and *Mischocyttarus basimacula*, N = 14) and two species of stingless bee (*Scaptotrigona subobscuripennis*, N = 144, and *Tetragonisca angustula*, N = 157) also had their net electrostatic charge measured.

**Fig. 2.**
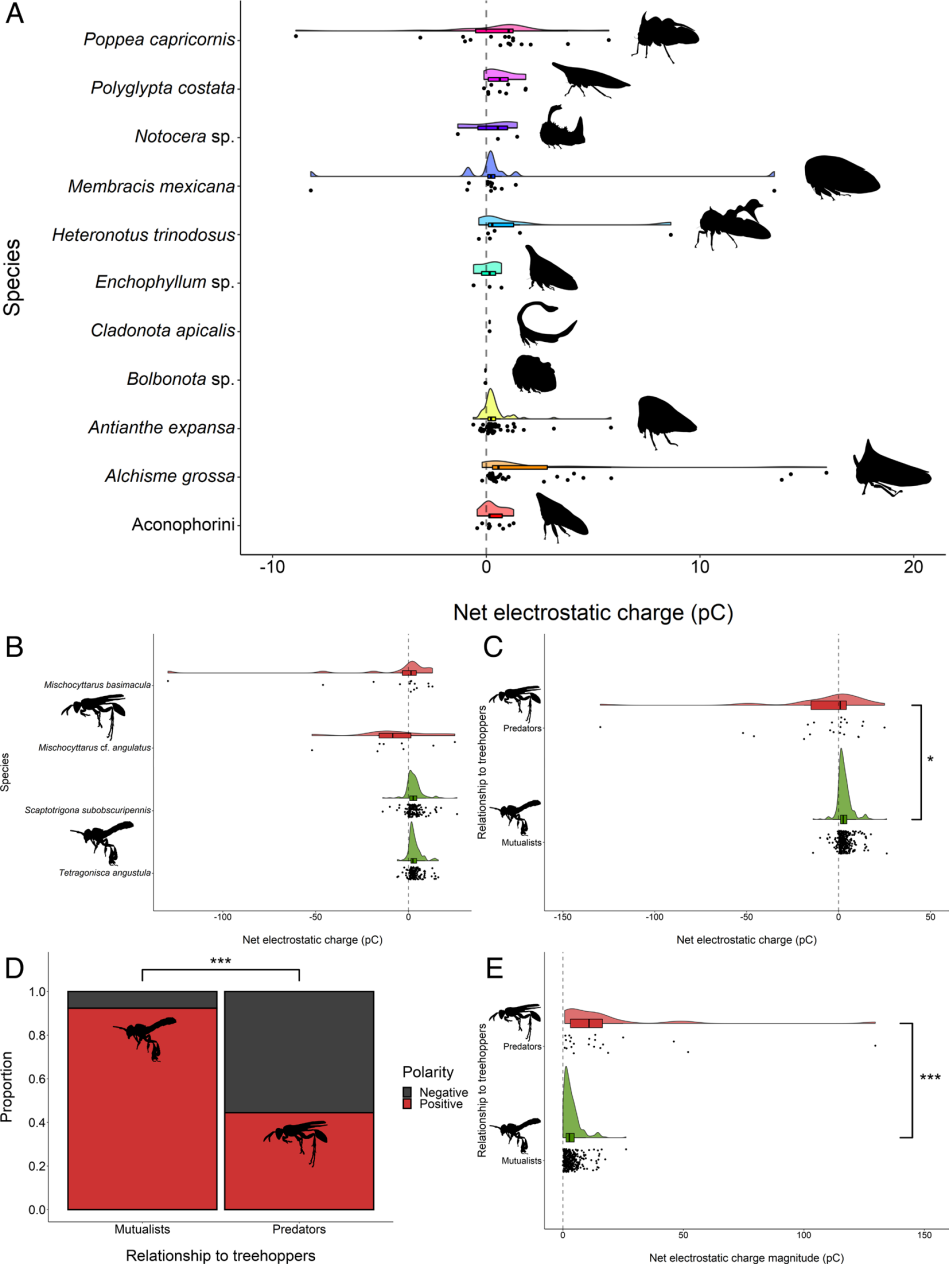
The net electrostatic charges measured on treehoppers (N = 151), predatory wasps (N = 22), and mutualist bees (N = 301). For raincloud plots, points indicate individual measurements; box and whisker show median, lower, and upper quartiles, and range; half-violin shows distribution of each dataset. Vertical dashed lines signify transition from negative to positive charge polarity. Asterisks denote statistically significant differences (*P < 0.05, ***P < 0.001). Please note different axes scales between plots. (*A*) Net charges of treehoppers. Clades include *P. capricornis* (N = 18), *P. costata* (N = 9), *Notocera* sp. (N = 3), *M. mexicana* (N = 13), *H. trinodosus* (N = 6), *Enchophyllum* sp. (N = 3), *C. apicalis* (N = 1), *Bolbonota* sp. (N = 1), *A. expansa* (N = 55), *A. grossa* (N = 32), and Aconophorini (N = 10). (*B*–*E*) Charge characteristics of predatory wasp species (red) including *Mischocyttarus basimacula* (N = 14) and *Mischocyttarus* cf. *angulatus* (N = 8), and mutualist bee species (green), including *Scaptotrigona subobscuripennis* (N = 144) and *Tetragonisca angustula* (N = 157). (*B*) The net electrostatic charge for individuals of each species. (*C*) The net electrostatic charge of individuals grouped by relationship to treehoppers. (*D*) The proportion of positive (red) versus negative (black) net electrostatic charge polarities for individuals grouped by relationship to treehoppers. (*E*) The net electrostatic charge magnitude of individuals grouped by relationship to treehoppers.

All of the treehopper individuals across the 11 species measured carried a nonzero net electrostatic charge (N = 151) (Fig. 2*A*), although compared to other animals measured previously, their net charges are relatively low. The majority of these charges were positive in polarity (n = 127, 84%), as opposed to negative (n = 24, 16%). The average net charge across all species was +0.92 ± 2.82 pC (mean ± SD), and the median net charge was +0.31 pC, with the maximum charge of +15.91 pC carried by an *A. grossa* individual, and the minimum charge of −8.91 pC carried by a *P. capricornis* individual. The mean net charge magnitude across all species was 1.32 ± 2.66 pC. The mean net charge magnitudes by taxa were Aconophorini = 0.49 ± 0.43 pC; *A. grossa* = 2.46 ± 4.27 pC, *A. expansa* = 0.52 ± 0.90 pC, *Enchophyllum* sp. = 0.49 ± 0.29 pC, *H. trinodosus* = 1.87 ± 3.36 pC, *M. mexicana* = 2.07 ± 4.06 pC, *Notocera* sp. = 1.11 ± 0.49 pC, *P. costata* = 0.75 ± 0.71 pC, and *P. capricornis* 2.04 ± 2.17 pC. The individuals measured for *Bolbonota* sp. and *C. apicalis* carried net charge magnitudes of 0.04 pC and 0.15 pC, respectively.

Each of the individual bees and wasps carried a nonzero net electrostatic charge (Fig. 2*B*). For simplicity, we treated sympatric wasps as predators due to vespids typically being predators of treehoppers (18, 29, 38–45) and sympatric stingless bees as mutualists of treehoppers due to meliponines being known protectors of treehoppers (18, 49–51). The mutualist stingless bee species *Tetragonisca angustula* (N = 157) carried a mean net electrostatic charge of +3.26 ± 3.41 pC, and a median net charge of +2.43 pC, with a maximum of +16.11 pC and a minimum of −5.99 pC. Their mean net electrostatic charge magnitude was 3.54 ± 3.11 pC. The majority of this species were positively charged (n = 147, 94%). The other mutualist stingless bee species *Scaptotrigona subobscuripennis* (N = 144) carried a mean net electrostatic charge of +3.05 ± 4.46 pC, and a median net charge of +2.57 pC, with a maximum of +26.23 pC and a minimum of −13.88 pC. Their mean net electrostatic charge magnitude was 3.71 ± 3.92 pC. The majority of this species were positively charged (n = 131, 91 %). The predatory wasp species *Mischocyttarus* cf. *angulatus* (N = 8) carried a mean net electrostatic charge of −8.17 ± 22.97 pC, and a median net charge of −8.47 pC, with a maximum of +25.03 pC and a minimum of −51.98 pC. Their mean net electrostatic charge magnitude was 17.79 ± 15.57 pC. The majority of this species were negatively charged (n = 6, 75 %). The other predatory wasp species *Mischocyttarus basimacula* (N = 14) carried a mean net electrostatic charge of −10.63 ± 37.38 pC, and a median net charge of +1.39 pC, with a maximum of +12.95 pC and a minimum of −129.63 pC. Their mean net electrostatic charge magnitude was 17.80 ± 34.32 pC. The majority of the individuals of this species were positively charged (n = 10, 71%). Overall, the net electrostatic charge was significantly different for the predatory species compared to the mutualist species (U = 4,224, *P* = 0.031) (Fig. 2*C*). These differences are manifested in both the charge magnitude (Fig. 2*E*) and charge polarity (Fig. 2*D*). Specifically, the net charge magnitude was significantly higher for predatory species than for mutualist species (U = 1,679.5, *P* < 0.001) (Fig. 2*E*). Predatory species were also significantly more likely than mutualist species to possess a negative charge polarity as opposed to a positive polarity (Fisher’s exact test, *P* < 0.001) (Fig. 2*D*). There was no significant difference in the relative humidity (U = 3,610, *P* = 0.479) or temperature (U = 3,288, *P* = 0.957) between the measurements of predators and mutualists.

### The Extreme Morphology of Treehoppers Increases the Strength of Electrical Stimuli.

**Fig. 3.**
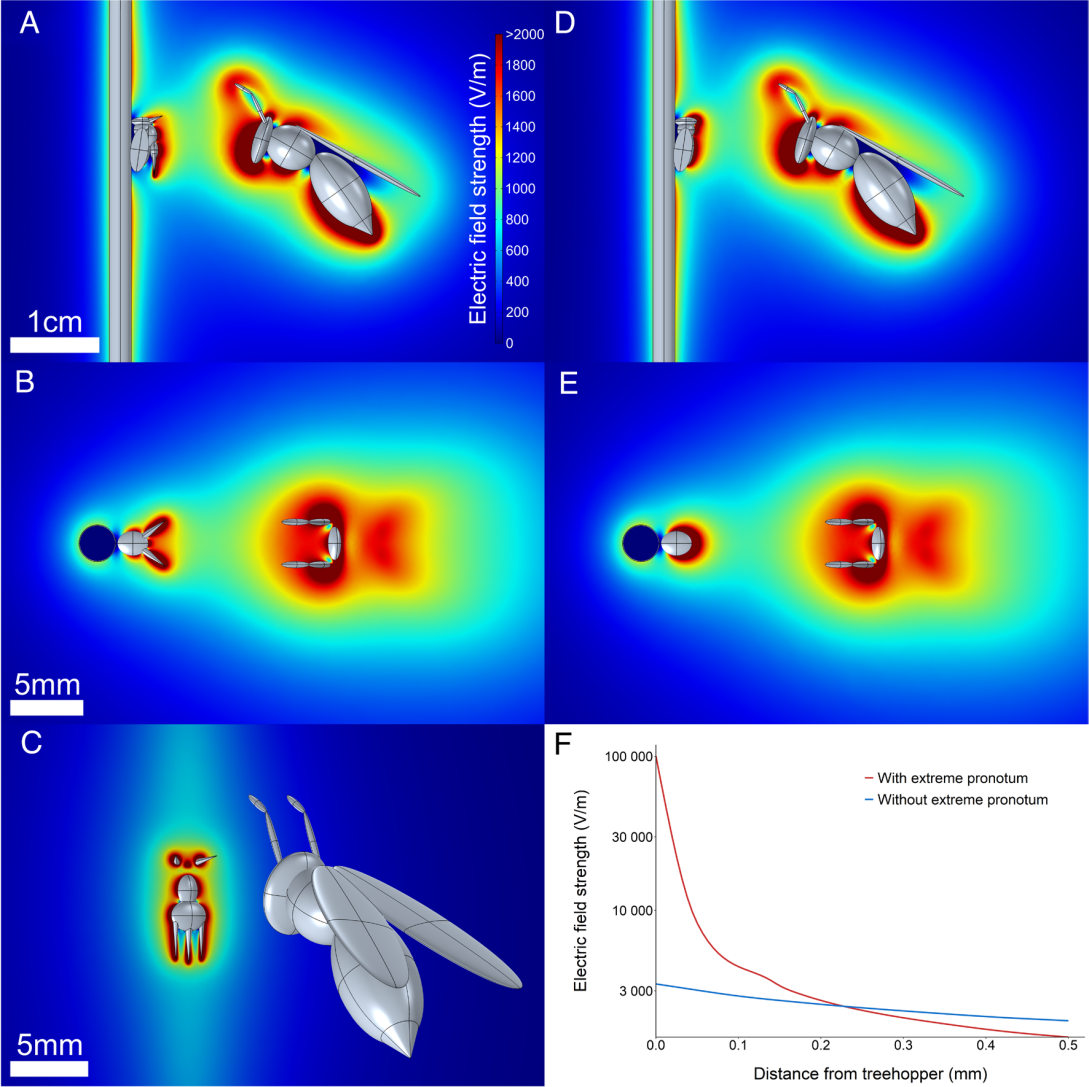
Three-dimensional finite element analysis model of the electric field between a typically charged *P. capricornis* treehopper with and without its extreme pronotum, situated on a plant stem, being approached by a typically charged predatory wasp, viewed from various angles. Color indicates electric field strength, truncated at 2 kVm^−1^ for clarity. Gray indicates three-dimensional model geometry. The treehopper representation is assigned the mean net charge magnitude measured for *P. capricornis* (2.04 pC), the wasp representation is assigned the mean net charge measured for *Mischocyttarus basimacula* (−10.63 pC), the plant stem is defined as electrical ground. (*A*–*C*) Lateral view, anterior view, and dorsolateral view of treehopper with extreme pronotum, descending, respectively. (*D* and *E*) Lateral view and anterior view of a hypothetical treehopper without extreme pronotum. (*F*) Electric field strength within 0.5 mm of the treehopper, with (red) and without (blue) *an* extreme pronotum. Derived from cutlines though the models (beginning from tip of suprahumeral horn for treehopper with extreme pronotum, and from center of the upper surface of the thorax for treehopper without extreme pronotum). Note log-scale *y*-axis. Lines smoothed using generalized additive models.

Then, based on these measurements, we calculated the strength and structure of the electric field that would exist between a typically charged treehopper on a plant and an approaching typically charged predatory wasp. These calculations were performed computationally in three dimensions using the finite element method. The representation of a treehopper was given the measured mean charge magnitude of the treehopper *P. capricornis*, and the wasp representation was assigned the mean charge measured for the predatory wasp *Mischocyttarus basimacula.* The resultant model shows that nonnegligible electric fields are produced when the two are in close proximity, with a magnitude in excess of 2 kV/m (Fig. 3). It is also seen that at points of extremity in the treehopper morphology, in particular, the distal features of the pronotum with high aspect ratios, the electric field is especially strong, rising to over 100 kV/m. By comparing the electric field around the treehopper with a real extreme morphology (Fig. 3 *A*–*C*), to the hypothetical treehopper without extreme morphology (Fig. 3 *D* and *E*), we see that the electric field is greatly modified in both geometry and strength by the extreme pronotum (Fig. 3*F*). Impressively, in this case, the electric field in the first 0.1 mm around the extreme pronotum increases by two orders of magnitude to over 100 kV/m, compared to approximately 3 kV/m around the nonextreme pronotum. This will greatly increase the stimulus strength available to any putative electroreceptors positioned on the pronotum, which are typically located within the first 0.1 mm. Such high electric field strengths experienced by mechanosensory hairs distributed across the pronotum will likely better facilitate the detection, locating, and identification of charged objects in the environment (57, 58). Importantly, our modeling likely even underestimates the electrosensory benefit of the extreme treehopper pronotum, because our models kept the same total charge on both treehoppers, and so the charge density on the nonextreme pronotum, with its smaller surface area, is artificially increased. Thus, overall, the extreme pronotum of treehoppers offers them potential increases in electrical sensitivity in a fivefold manner: 1, by increasing the magnitude of electric fields around putative sensory structures due to geometry (Fig. 3*F*); 2, by increasing the total surface area available for charge to deposit, also increasing the electric field strength around them; 3, by increasing the total surface area available to electrosensory structures, so that more sensors can exist; 4, by reaching out into the environment, closing the gap to external electric field sources and thus increasing the electric field strength in ecological interactions via proximity; and 5, by offering a complicated morphological base with sensors disparately arranged in position and angle, allowing for greater ease in the differential measurements required for electrolocation. Interestingly, we see that after 0.2 mm, the electric field strength is actually lower for the treehopper with an extreme pronotum. The ecological consequence of this is that the treehopper pronotum may actually decrease the electrical detectability of the treehoppers to any electroreceptive predators, a form of electrical crypsis, while increasing the sensitivity of the treehoppers themselves.

### Treehoppers Retreat from Electric Fields.

**Fig. 4.**
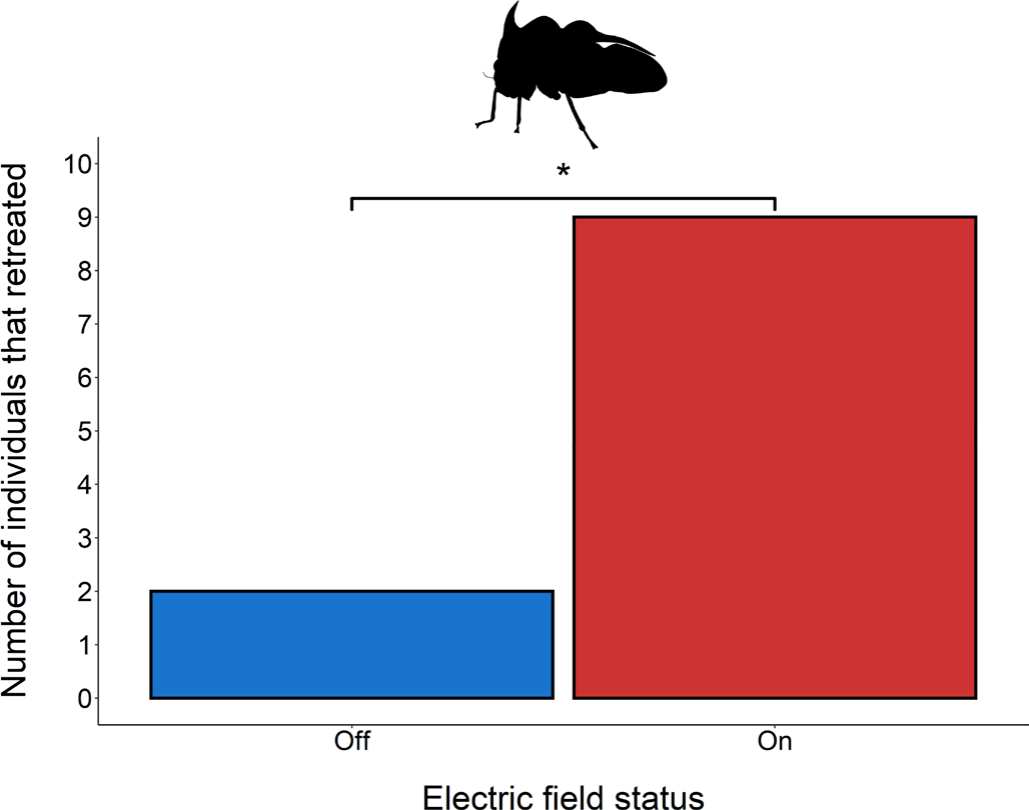
Number of *Poppea capricornis* treehoppers that retreated from the summit of a wooden pole when exposed to treatment conditions (750 V voltage on, red, n = 20) and control conditions (voltage off, blue, n = 20), total N = 40. Asterisk (*) denotes a statistically significant difference of *P* < 0.05.

It was also important to test whether treehoppers respond behaviorally to electric fields and thus are capable of electroreception. This was done by comparing the behavioral responses of adult *P. capricornis* (N = 40) exposed to treatment electrical conditions (electric field on, n = 20), and control electrical conditions (electric field off, n = 20). In the treatment group, treehoppers were exposed to a 1.5 cm diameter spherical electrode held at 750 V, 1 cm away, switched on as the treehopper summited a wooden pole. In the control group, conditions were otherwise identical, but the electrode was disconnected from the voltage source. Each trial was filmed and subsequently analyzed blind to treatment/control conditions. Treehoppers were significantly more likely to retreat from the electrode when the electric field was on (n = 9, 45%) compared to when it was off (n = 2, 10%) (Fisher’s exact test, *P* = 0.031) (Fig. 4). There was no significant difference in the relative humidity (U = 201, *P* = 0.989) or temperature (U = 192.5, *P* = 0.849) between the treatment and control groups, and therefore, the behavioral differences between the two groups can be attributed to the presence or absence of the electric field.

### The Pronotum of Treehoppers Is Covered in Sensory Structures That Respond to Electric Fields.

**Fig. 5.**
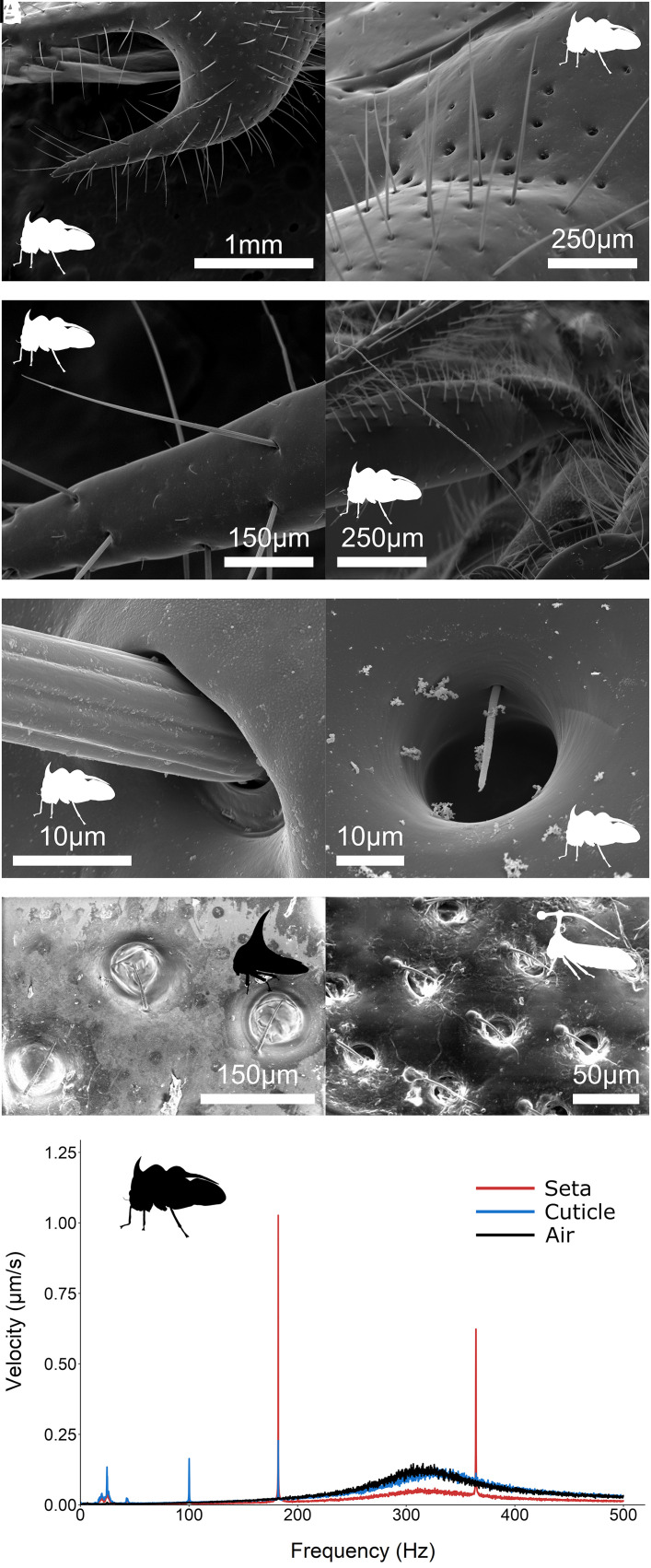
Scanning electron microscopy images of the various candidate electroreceptors found on treehoppers and their electromechanical response. (*A*) Distribution of the erect-type setae on the distal regions of the pronotum of *P. capricornis*. (*B*) Distribution of the pit-type setae on the proximal regions of the *P. capricornis* pronotum, with erect-type setae in the foreground. (*C*) Closer view of the erect-type setae of *P. capricornis*. (*D*) The reduced antenna of *P. capricornis*, found on the face (anterior head surface) of the insect. (*E*) Closer view of an erect-type seta on the pronotum of *P. capricornis*, showing the articulation in the cuticle of the pronotum. (*F*) Closer view of a pit-type seta on the pronotum of *P. capricornis*. (*G*) The pit-type setae found on the pronotum of *Umbonia crassicornis*. (*H*) The pit-type setae found on the wing of *Bocydium globulare*. (*I*) Electromechanical responses of the pronotal erect-type setae of *P. capricornis* (red), the surrounding cuticle (blue), and the surrounding air (black), to a 182 Hz sinusoidal electric field, as measured by laser Doppler vibrometry. Each line represents mean response of five individuals (N = 5).

To begin to identify the site of electroreception in treehoppers, 52 individuals from 20 species were examined under light and electron microscopy. Several candidate electroreceptors were identified. The pronotum is well endowed with setae that appear to be articulated and thus mechanosensory (Fig. 5) in concordance with observations in other treehopper species (35–37). These exist in two main types: longer setae projecting perpendicular to the cuticle, termed here “erect-type” (Fig. 5 *A*, *C*, and *E*), and shorter setae protruding from near to the rim of pits in the cuticle and projecting across some or all of the diameter of the pit, termed here “pit-type” (Fig. 5 *B*, *F*, *G*, and *H*).

While the distribution and relative abundance of each of these seta types varies at the species level, some general trends can be identified. First, the distribution of both erect-type and pit-type setae is not uniform across the pronotum of individual treehoppers. Pit-type setae are found far more frequently on the proximal regions of the pronotum, especially near the face, whereas erect-type setae are predominantly found at distal regions of the pronotum, particularly at points where the cuticle changes direction suddenly, such as along the convex edge of ridges, or at the tip of spines and spikes. The pits associated with the pit-type setae become wider and shallower in a relatively continuous spectrum, the more distally they are found along the pronotum, eventually disappearing completely. At this point, the hairs associated with these pits become erect-type hairs; longer and protruding perpendicular to the cuticle. It therefore seems very likely that both seta types are homologous and share developmental characteristics. Pit-type setae were additionally found on the wings of some species (Fig. 5*H*). It is also worth noting that treehoppers possess small reduced antennae on the front of their head (Fig. 5*D*) that could also be electroreceptive, as has been shown for the antennae of honeybees (11). All of these structures appear to be mechanosensory, and thus electrosensory if actuatable by external electric fields.

Such possible electrosensory capabilities were probed further using laser Doppler vibrometry (LDV) measurements on *P. capricornis*. In every individual tested (N = 5), the erect-type setae on the pronotum responded mechanically to a 182 Hz sinusoidal electric field, mimicking the frequency component of a wasp wingbeat (Fig. 5*I* and *SI Appendix*, Fig. S3). Deflection velocities of the setae were much greater than the surrounding cuticle, showing that it is the setae, and not the pronotum as a whole, that are electromechanically actuated by the electric fields.

### The Pit-Type Setae of Treehoppers Convey Specialized Benefits for Electrical Sensitivity.

To examine the putative electroreceptive biophysics of the different setae identified in treehoppers, and explore for any differences or specializations in their electrical sensitivity, numerical mathematical calculations of the electromechanical sensitivity of both the erect-type and pit-type mechanosensory setae were conducted. This involved two-dimensional computation of their electrical sensitivity contours, utilizing the mathematical principles and techniques described in detail in previous theoretical work (59).

**Fig. 6.**
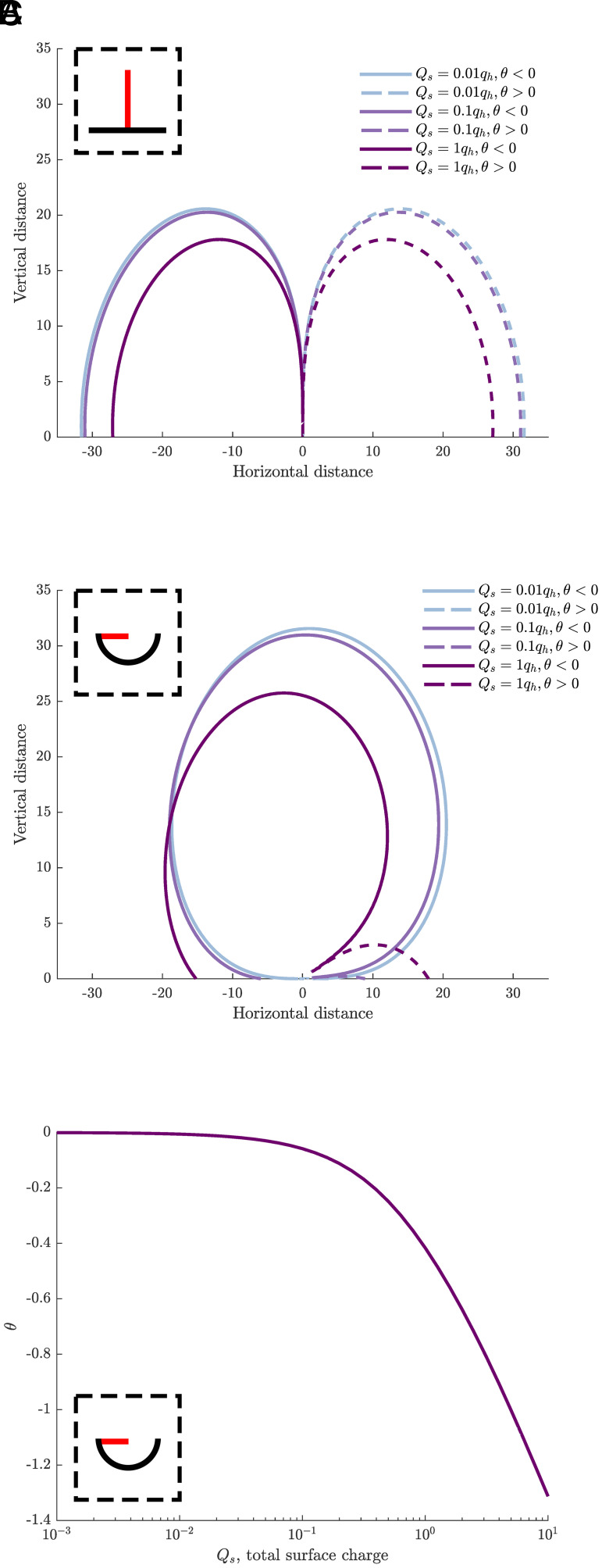
Numerically derived sensitivity contours for the erect-type setae (*A*) and pit-type setae (*B*) found on the treehopper pronotum. Boxes show approximations of sensilla geometry with setae colored red and cuticle in black. Threshold defining sensitivity contours is set to an angular deflection (θ) of 0.001 rad, caused by an external point charge, q_p_ equal in magnitude but opposite in polarity to the charge on the seta tip, q_h_. Distances are given relative to seta length. Q_s_ is the total charge on the surrounding cuticle, relative to q_h_. Dashed lines denote positive angular deflection, solid lines denote negative angular deflection. Line colors indicate varying relative charging between cuticle and seta. (*C*) Numerically derived relation for the angular deflection (θ) of a pit-type seta as the total charge of the pit cuticle, Q_s_, increases relative to the charge on the seta tip q_h_.

The mathematically produced sensitivity contours for the erect-type setae concur with those produced in previous analyses (59), however, here we include a charge on the cuticle, not previously considered. We see across both seta types that as the total charge on the cuticle increases relative to the charge on the seta, the range of sensitivity decreases (Fig. 6). This is due to the like-charges of the cuticle and seta repelling each other, increasing the force required to deflect the seta from its equilibrium position toward the cuticle. Overall, in both cases, the same approximate range of sensitivity is covered. However, the sensitivity contours for the pit-type setae are noticeably different from those of the erect-type setae in several aspects. Most notably, there is a blind spot in the sensitivity contours of the erect-type setae directly above the sensilla (Fig. 6*A*) that is not present in the pit-type setae (Fig. 6*B*). Furthermore, the pit-type setae are only significantly deflected in one direction for each polarity, independent of the location of the stimulus charge. Last, there is a strong coupling in the pit-type setae between the charge of the cuticle and the deflection of the seta, wherein the surface charge of the cuticular pit will deflect the seta from its equilibrium position, i.e., the position of the seta if there were zero charge on the cuticle, even at relatively low cuticular surface charges (Fig. 6*C*).

## Discussion

The results presented in this study suggest that not only are treehoppers capable of detecting the electric fields emitted by their predators but that the morphology of the pronotum in many species likely increases their sensitivity to such electrical stimuli.

First, we have shown that treehoppers and the mutualists and predators with which they interact are capable of accumulating a net electrostatic charge despite the high relative humidity of their environments. These are the first charge measurements reported for tropical organisms in situ and so confirm that the principles of electric ecology established in temperate environments (1, 9, 16, 56, 60) can be extended to the tropics (10).

Importantly, we document that the net electrostatic charge of predatory wasps is significantly different from mutualist bee species, both in terms of magnitude and polarity. These qualities could be seen as electrical analogs to the “loudness” and “flavor” of their electric cues respectively. Therefore, electroreceptive insects, such as treehoppers, have the information available to them in their environment to be able to potentially discern predators from mutualists, based on electrical cues alone. As such, if the electroreceptors of treehoppers are independently or collectively capable of determining charge magnitude or polarity, they may use electroreception to prevent falsely identifying mutualists as predators and vice versa, improving the efficacy and efficiency of both their predator defense strategies and mutualistic interactions. It is important to note, however, that some species of wasp have also been witnessed to form mutualisms with treehoppers (49), and thus, the use of electroreception for threat identification would likely also require the integration of other contextual cues. Furthermore, given the variations in charge and the overlap of the distributions observed for each species, identification of predators versus mutualists by electrical means alone could be prone to error and would thus benefit from incorporation into a multimodal sensory assessment.

When modeling the electric field generated between a typically charged predatory wasp situated within centimeters of a typically charged treehopper, we show that it reaches a magnitude already reported to be detectable by other arthropods (9, 12). Therefore, sufficient electrical information is present during this interaction for treehoppers to potentially utilize it as a cue.

The behavioral data suggest that electric potentials of the magnitude reported (1, 9, 16, 61–65) for clades known to predate upon treehoppers (18, 29, 38–45) elicit a retreating behavior in *P. capricornis* treehoppers that is a proxy for threat perception. As such, *P. capricornis* and potentially many other treehopper species are likely capable of using electroreception for predator detection. It is important to note here that the DC electric field utilized as the stimulus in treatment behavioral trials lacked temporal components that would make the stimulus more akin to a real predatory cue (9). Therefore, it is probable that treehoppers would exhibit even greater degrees of electric sensitivity if provided with a more ecologically specific stimulus. Furthermore, treehoppers are known to utilize a sophisticated vibrational communication system during predator attacks (38, 45, 66–68). Future studies could look to eavesdrop on these vibrational communications and test their emission or suppression in response to electrical stimuli.

Although significantly different, the typical charges of the species that form mutualisms with treehoppers are still in the same order of magnitude as predators, and therefore, it is also very plausible that treehoppers detect and respond behaviorally to the electric fields of approaching mutualists too. To extend the horizons of our knowledge of the ecological role of electricity and electroreception, behavioral studies should investigate whether mutualistic interactions such as these are also mediated by static electricity and its detection. Similarly, parasitoids, particularly parasitoid wasps, are also critical natural enemies of treehoppers (18, 40, 69, 70), therefore, treehoppers would also benefit from using electroreception to detect these threats. While charge measurements remain to be made of species that parasitize treehoppers, it is probable that they carry net electrostatic charges akin to their nonparasitoid relatives and therefore could likely be detected by the same mechanisms shown in this study. Future work should seek to test this hypothesis.

The morphological examinations concur with previous studies showing that treehoppers possess a diverse array of mechanosensory structures, particularly on the pronotum. As these structures have previously been shown to be innervated (35, 36, 44), and mechanosensory setae and antennae are known to be electroreceptive in other taxa (8, 9, 11, 12, 17), it is thoroughly probable that the identified structures are responsible for electroreception in treehoppers. Indeed, the LDV conducted in this study shows a clear electromechanical response of the erect-type pronotal setae to an external electric field. The pit-type setae likely respond too. When exposed to a 182 Hz sinusoidal electric field, congruent with the wingbeat frequency of the predatory wasp *Mischocyttarus basimacula* (*SI Appendix*, Fig. S3), the setae oscillate strongly at both the fundamental frequency and the 2nd harmonic. This informs us that the electrostatic actuation of the setae is acting in a phasic manner, more akin to bumblebee setae (17, 71), rather than in a tonic manner, as in spiders (12) and caterpillars (9). It should be noted though that this may be an artifact of the fact that this LDV was performed on deceased and partially dehydrated treehoppers that may have reduced conductivity and/or stiffness.

The numerical mathematical calculations of the sensitivity contours for both the erect-type and pit-type setae reveal that their electrical sensitivities, and thus modes of operation, are fundamentally different. First, the pit-type setae do not exhibit the same electrosensitivity blind spot directly above the sensilla. This indicates that these pit-type setae convey a greater efficacy as electroreceptors by maintaining electrosensitivity to stimuli coming from a wider range of angles. Further, the observation that the pit-type setae will be predominantly deflected in a single direction by each polarity, irrespective of the charge location, means that excitingly, even an animal with a single pit-type seta could theoretically be capable of determining the polarity of an external charge source, if it has a means of deducing the direction of deflection in that particular seta. This suggests that the discrepancy in charge polarity seen between predatory and mutualist hymenopterans could indeed be a detectable cue utilized by treehoppers for identification. Although this would rely upon the treehopper having some implicit knowledge of the charge polarity of the seta, this polarity could most likely be assumed by the treehopper because the measurements made in this study show that most treehopper species consistently exhibit a positive net charge polarity. The pit-type setae also exhibit a strong coupling between the surface charge within the pit and the deflection of the setae. The biophysical consequence of this is that if the cuticle is readily polarizable [i.e., if a charge separation or dielectric polarization can be induced, as in ticks (60)], the pit may secondarily convey electrical information to the seta, wherein the cuticle is polarized by an external electrical stimulus, and subsequently this polarization deflects the seta. Thus, the electric field could be sensed not only via direct electrostatic actuation by the electric field between the seta and stimulus source but also, indirectly, via deflection of the seta resultant from interaction with a dynamically polarized cuticle. These electromechanical characteristics unique to the pit-type setae suggest that they may have evolved in part to provide better sensitivity for electroreception.

Furthermore, the finite element analysis illustrates that the electric field between a treehopper and a charged predator is strongest around the features of highest geometric extremity on the pronotum, meaning that these structures are effectively acting analogously to an electrostatic lens. This supports the hypothesis that the various pronotal protrusions exhibited by the majority of treehopper clades will act to increase the sensitivity of pronotal mechanosensory hairs to external electric stimuli. Additionally, this electrostatic lensing effect may explain the tendency of the erect-type setae to be more frequently located on the distal and more geometrically extreme regions of the pronotum, such as ridges and spines, where the electric field is strongest. Furthermore, erect-type setae located at the tip of ridges or spines will not experience the electrostatic damping identified in Fig. 6*A*, because the surface charge of the cuticle is no longer perpendicular to the setae, and as such positioning erect-type setae upon these features may improve their electrical sensitivity.

Variations in environmental and ecological conditions are thought to lead to diversification in evolutionary adaptations of sensory systems and their associated morphology, subsequently leading to speciation (72–81). If this is also the case for electroreception, specialization within the electroreceptive sense could have contributed to the diversification of adaptive radiations, such as that seen in the treehoppers. Other animal clades exhibiting morphological diversity or extremity, for example, the numerous clades of other true bugs, beetles, and spiders with extreme protrusions, many of which are currently without functional explanation, should also now be examined, in light of the realization herein that morphological extremity and adaptive radiations could potentially be driven by specializations of the electroreceptive sense.

It is important to note that electroreception is almost certainly not the only function of the extreme morphologies seen in treehoppers. As mentioned previously, various possible visual functions such as crypsis, masquerade, mimicry, and visual signaling, as well as physical defense have also been suggested (26–32). Each of these likely contribute to the evolution of the pronotum in specific treehopper clades. Furthermore, other sensory modalities such as chemoreception, substrate vibration detection, air flow sensing, and acoustic detection may also be conferred or augmented by the pronotum, though the relationship between these senses and pronotum morphology remains to be explored. All of these potential functions, including electroreception, likely interact with one another when influencing the morphological evolution of treehoppers in ways unique to each clade, either through synergistic selection or selection trade-offs.

In conclusion, this study provides evidence of a ubiquitous sensory function for the treehopper pronotum, demonstrating that morphological features across multiple scales will heighten electroreceptive sensitivity. In doing so, we introduce the possibility that electroreception may contribute to the evolution of morphological extremity and diversity.

## Materials and Methods

### Animal Collection and Care.

Treehoppers were wild caught from various sites around Estación Biológica La Selva, Heredia province, Costa Rica; San Isidro, Heredia province, Costa Rica; and Ciudad Universitaria Rodrigo Facio, San Pedro, San José province, Costa Rica. Treehoppers were housed together in mesh enclosures (≈30 × 30 × 30 cm), with water-potted cuttings of their host plants. After inclusion in an experiment, individual treehoppers were rehoused in a different enclosure to prevent resampling. Stingless bees and wasps were measured in situ at their natural nesting sites around San Pedro and San José, Costa Rica. Voucher specimens were collected for each species to allow for subsequent identification.

### Charge Measurements.

Charge measurements were made using a previously described ring electrode (inner diameter = 7.7 cm, width = 2.5 cm) system connected to a custom-built picoammeter, feeding into a digital acquisition system (NI USB-6009, National Instruments, Austin, TX, USA), and recorded in MATLAB R2018a (MathWorks, Natick, MA, USA) at a 1,000 Hz sampling frequency (9, 56).

Wasps and bees had their charges measured while freely flying to and from their nests at various sites around San Pedro and San José, by mounting the ring electrode with a tripod and/or electrical insulating tape near their nests. For stingless bees, the ring electrode was mounted around the clearly defined nest entrances. For paper wasps, which build open nests, the ring electrode was mounted near the side upon which the majority of colony members were situated, to maximize the chance of individuals flying through the ring upon departure and arrival. An observer watched the nest for the entirety of the recording period, noting the timings of when bees or wasps passed through the loop. Only clean passthroughs, wherein no contact was made by the insect with the ring electrode system, were included in the analysis. Two species of mutualist bees (*Scaptotrigona subobscuripennis*, N = 144, and *Tetragonisca angustula*, N = 157) and two species of predatory wasp (*Mischocyttarus* cf. *angulatus*, N = 8, and *Mischocyttarus basimacula*, N = 14) had their net electrostatic charge measured.

Treehoppers had their charges measured by encouraging them to walk onto a bamboo stick, upon which they were allowed to walk at least one length of the stick (30 cm), before they would freely jump or fly away through the ring electrode mounted at the end of the stick. Individuals that did not readily jump or fly through the ring electrode voluntarily were instead flicked through the ring electrode by brief contact with a secondary bamboo stick. A total of 151 individual treehoppers from 11 different evolutionary clades had their net electrostatic charge measured, namely *P. capricornis* (N = 18), *P. costata* (N = 9), *Notocera* sp. (N = 3), *M. mexicana* (N = 13), *H. trinodosus* (N = 6), *Enchophyllum* sp. (N = 3), *C. apicalis* (N = 1), *Bolbonota* sp. (N = 1), *A. expansa* (N = 55), *A. grossa* (N = 32), and Aconophorini (N = 10). An example of the voltage-trace produced by the charge measurement system for a treehopper is shown in *SI Appendix*, Fig. S1. The temperature and relative humidity were recorded for all charge measurements to monitor and subsequently test for any significant variance in the environmental conditions between measurement sessions and locations.

### Computational Modeling of Electric Fields.

All computational models of the electric fields in nature and the experimental apparatus were produced using three-dimensional finite element analysis in COMSOL Multiphysics^®^ v. 5.4 (COMSOL AB, Stockholm, Sweden). All models were created using either the Electrostatics or Electric Currents interfaces, as appropriate, within the AC/DC module. The Electrostatics interface requires the relative electric permittivity, ε_r_, to be defined for each material, whereas the Electric Currents interface requires both ε_r_ and the electrical conductivity, σ, to be defined for each material. Complete details of the electrical properties ascribed to each material in the models, along with their sources, can be found in *SI Appendix*, Table S1. All data from the models are presented as two-dimensional slices through the three-dimensional models. Cut lines were utilized to extract the electric field strength at precise regions of interest.

For the model of the electric field between a predatory wasp and a treehopper, the electrostatics interface was used. The model was contained within a 0.15 × 0.15 × 0.15 m cube. At the center of this cube a 0.15 m long cylinder with a diameter of 2.5 mm was placed, oriented vertically, and bridging the top and bottom surfaces of the cube, representative of the stem of a plant. This cylinder was assigned the material properties of “Plant tissue.” Midway up this cylinder, a coarse three-dimensional representation of a *P. capricornis* treehopper was positioned, comprising intersecting ellipsoids and cones, based on scale measurements of a voucher specimen. In addition, a three-dimensional representation of a wasp, also comprising intersecting ellipsoids and cones, was constructed. The center of the wasp representation’s thorax was positioned approximately 1.875 cm away from the center of the plant stem cylinder, with its head roughly centered on the middle of the treehopper representation. This geometry was meshed under “extremely fine” parameters, with the minimum element size then reduced further to 0.01 mm. The two animal representations were both assigned the material properties of “Insect tissue.” The remainder of the model was assigned the material properties of “Air.” The surface of the wasp representation was assigned a charge of −10.63 pC, while the surface of the treehopper representation was assigned a charge of +2.04 pC, based on the means of the charge and charge magnitude measurements made in this study for *Mischocyttarus basimacula* and *P. capricornis,* respectively. The surface of the plant stem cylinder was defined as the electrical ground (7, 9, 12, 16, 56, 60, 82–84). A variation of this model was produced that was otherwise identical, except for containing a modified treehopper geometry that removed all of the expanded features of the pronotum, for purposes of comparison. The cutlines for examining the electric field strength around each treehopper geometry were 0.5 mm long and placed perpendicular to direction of the plant stem. For the extreme, realistic, treehopper geometry, this cutline began at the tip of one of the suprahumeral horns. For the hypothetical treehopper geometry without extreme features, the cutline began at the center of the upper surface of the thorax.

The model of the electric fields within the behavioral experiment apparatus (*SI Appendix*, Fig. S2) was built using the Electric Currents interface. The model was contained within a 0.6 × 0.6 × 0.6 m cube. At the center of this cube, flush to the bottom surface was placed a 0.165 × 0.1 × 0.04 m cuboid, representing the silicone rubber block from the behavioral apparatus. Placed directly through the center of this cuboid was a 30 cm long cylinder with a diameter of 3 mm, representative of the bamboo stick penetrating the full depth of the silicone. Directly to the side of the tip of this cylinder, a sphere was placed with a diameter of 1.5 cm, representing the spherical electrode in the behavioral apparatus. The gap between the surface of the sphere and the center of the cylinder was exactly 1 cm. One side of the sphere had a 1.5 mm minor segment removed from it, and upon the resultant flat surface was attached a 15 cm long cylinder with a diameter of 1 cm, representative of the wooden support in the behavioral apparatus. All the dimensions listed here were derived from measurements of the actual behavioral apparatus. The silicone block representation was assigned the material properties of “Silicone,” the central bamboo stick representation, and the wooden support representations were assigned the material properties of “Wood,” and the electrode representations were assigned the material properties of “Aluminium.” The remaining domains were assigned the material properties of "Air". The surface of the electrode representation was assigned a voltage of 750 V, while the bottom surface of the entire model was defined as electrical ground.

### Behavioral Experiments.

To test for the ability of treehoppers to detect electric fields, an experimental apparatus was assembled consisting of a bamboo stick (length = 30 cm, diameter = 3 mm) pushed into a 4 cm thick block of silicone, such that a 26 cm portion of wooden pole stood stably upright. A 1.5 cm diameter spherical conductive metal electrode was placed with its nearest point 1 cm from the pole, with its center level with the highest point of the pole. This electrode was electrically connected to a PS350/5000 V-25 W high-voltage power source (Stanford Research Systems, Inc., Sunnyvale, CA, United States). For each experimental trial, individual treehoppers (*P. capricornis*, N = 40) were picked up and placed below a line marked 3 cm above the silicone. Each treehopper was then allowed to climb the pole and upon reaching its summit, the high voltage output was switched on, outputting a DC voltage of +750 V, congruent with other studies on electric ecology and estimates of the typical surface potentials of animals (1, 9, 10, 12, 13, 16, 60–65, 85). In treatment trials (n = 20), this voltage was connected to the spherical electrode, whereas in control trials (n = 20), this voltage was not connected to the electrode and the electrode was allowed to electrically float. The electric fields produced by this experimental apparatus can be seen in *SI Appendix*, Fig. S2. Trials were alternated between treatment and control conditions in an ABBA design, and the temperature and relative humidity were recorded for each trial to ensure that environmental and time-related conditions were consistent between the treatment and control groups. Each trial was filmed at 50 frames per second with a Nikon 3400 DSLR camera equipped with an AF-P DX NIKKOR 18 to 55 mm f/3.5 to 5.6G lens (Nikon Corporation, Tokyo, Japan). Videos were subsequently analyzed blind to whether or not a trial was under treatment or control conditions using BORIS (86), assessing whether or not each treehopper retreated from the summit of the pole within 20 s of voltage onset. A treehopper was deemed as retreating if it rotated around to face downward and began to descend the pole.

### Morphological Examinations.

Various treehopper species exhibiting a wide range of pronotal morphologies were examined under light microscopy and scanning electron microscopy (SEM) with the objective of identifying and characterizing the presence, morphology, and distribution of candidate electroreceptors on the treehopper pronotum. Light microscopy was conducted on a broad diversity of treehoppers consisting of 52 individuals from 20 different species, obtained from museum and private collections. For a full inventory of the species examined under light microscopy and their sources, see *SI Appendix*, Table S2. Light microscopy was performed with the use of a Leica MZ16 stereomicroscope (Leica Microsystems GmbH, Wetzlar, Germany), lit by a dual-source LED lighting system (Brunel Microscopes Ltd., Chippenham, United Kingdom). *P. capricornis* treehoppers intended for imaging by SEM were stored intact, being placed into 70% ethanol at the collection site for preservation. Prior to imaging, treehoppers were dehydrated further by moving them into 100% ethanol for 3 h and then placing them into a Leica EM CPD300 critical point dryer (Leica Microsystems GmbH, Wetzlar, Germany). After dehydration, treehoppers were sputter-coated with a gold palladium alloy using an Emitech K757X (Quorum Technologies Ltd., Ashford, United Kingdom). All SEM was performed with the use of an FEI Quanta 200 FEG scanning electron microscope (Field Electron and Ion Company, Hillsboro, OR, United States), utilizing a 20 kV accelerating voltage and an Everhart–Thornley detector. Museum specimens of *Umbonia crassicornis* and *Bocydium globulare* that could not be sputter-coated were instead examined as uncoated air-dried specimens using variable pressure electron microscopy on a Zeiss EVO 15 scanning electron microscope (ZEISS Group, Oberkochen, Germany), utilizing a 10 kV accelerating voltage and a variable pressure secondary electron detector.

### LDV.

LDV was conducted on *P. capricornis* treehoppers (N = 5) to assess for any electromechanical responses from candidate electrosensory structures. Prior to measurement, *P. capricornis* individuals stored in 70% ethanol were rehydrated up to 50% ethanol for a minimum of 24 h and then removed from solution immediately before measurement and glued by the legs and abdomen to a balsa wood platform. This platform was held by a clamp, magnetically attached to a metal antivibration table (TMC 784-443-12R; Technical Manufacturing, Peabody, MA, USA), situated in an acoustically isolated, semianechoic, and electrically shielded room. LDV was performed with a Polytec PSV500Xtra laser Doppler vibrometer, equipped with a PSV-A-410 close-up unit measuring head (Polytec GmbH, Waldbronn, Germany). Measurements were recorded in the PSV 9.3 acquisition software. The vibrometer range was set to a sensitivity of 2.5 mm s^−1^ V^−1^, with no tracking filter applied. The FFT measurement mode was used, with magnitude averaging over 10 samples per measurement, and 3,200 FFT lines with 0% overlap and a rectangular window. Electrical signals were generated within the software, and then amplified with a custom-built 40× voltage amplifier, with the positive connection outputted to a 1.5 mm diameter spherical silver electrode. A 182 Hz sine wave stimulus was utilized. This signal frequency was selected based on the measured wingbeat frequency of the predatory wasp *Mischocyttarus basimacula* measured in this study (183 ± 3 Hz). The signal was generated with a peak voltage of 8 V, increased to 320 V by the amplifier. For each individual, a scan comprising three single-point measurements was taken; an erect-type pronotal seta, the nearby cuticle, and the surrounding air. The nearby cuticle and air were measured for control purposes.

### Mathematical Calculations of Setae Electrical Sensitivity.

Previous calculations (59) were modified to apply to the erect-type setae of treehoppers by calculating the sensitivity contours for a rigid rod representative of the seta with a point charge located at its tip. This seta was positioned perpendicular to a line representing the cuticle, with its charge density represented by 25 point charges uniformly distributed symmetrically about the base of the seta. The pit-type setae sensitivity contours were calculated based on a horizontally oriented rigid rod with a point charge at its tip, representing the seta, protruding from the rim of one side of a semicircle, representing the pit cupule. The radius of the semicircle was equal to the length of the rod, such that the seta protruded halfway across the diameter of the pit. The 25 point charges were uniformly distributed across the length of the pit semicircle. All quantities in the mathematical calculations are dimensionless, in order to maintain validity across different scales. All length scales are given relative to the length of the seta, and all charge values are given relative to the magnitude of the point charge at the tip of the seta, with the total charge of the cuticle being the sum of the point charges along its length. The sensitivity contours were defined as all of the locations at which a point charge of −q_p_ (relative to the seta tip charge) deflects the setae to a minimum threshold, here 0.001 rad, as a result of Coulomb forces between the seta charge, stimulus charge, and substrate charges. This angular threshold of 0.001 rad is based upon the minimum angular deflection known to elicit a neural response in other arthropod mechanoreceptors (17, 59, 87–90). All computation was performed in MATLAB 2020b (MathWorks, Natick, MA, USA).

### Statistics.

All statistical analysis was conducted in R 4.2.1 (91). To test for any statistical differences in the net electrostatic charge between predatory wasps and mutualist bees, the four study species were grouped by their relationship to treehoppers as either “predators” or “mutualists” for analysis. All datasets were tested for normality visually with the use of Q–Q plots, and statistically with application of the Shapiro–Wilk test. Any datasets not meeting the normality requirements for parametric statistical tests were instead investigated with nonparametric statistical tests. Mann–Whitney *U* tests were used to compare the relative humidity and temperature at the time of measurement between the predator and mutualist groups to ensure that no significant difference existed in these environmental conditions that may have been a confounding factor influencing the charge measurements. Mann–Whitney *U* tests were also used to compare the net electrostatic charge and net electrostatic charge magnitude of predators and mutualists, using Levene’s test to assess the homogeneity of variance across the groups, which informs the interpretation of the Mann–Whitney *U* test. The relative frequency of negative and positive polarities between predators and mutualists was assessed with the use of Fisher’s exact test. The number of treehoppers that did or did not retreat in the behavioral experiments under treatment or control conditions was similarly compared with the use of Fisher’s exact test.

## Supplementary Material

Appendix 01 (PDF)

## Data Availability

Behavioral data, charge data, and LDV data have been deposited in Mendeley Data (10.17632/bwgwyzgcb7.1) (92).
