# Supplementary material for: Electroreception in treehoppers: How extreme morphologies can increase electrical sensitivity
Source: Proc Natl Acad Sci U S A. 2025 Jul 21;122(30):e2505253122. doi: 10.1073/pnas.2505253122 (PMC12318218; doi:10.1073/pnas.2505253122)
Supplement: Supplementary file 1 — Appendix 01 (PDF) [file pnas.2505253122.sapp.pdf]

PNAS

Supporting Information for
Electroreception in treehoppers: how extreme morphologies increase electrical sensitivity

Sam J. England[1,2]*, Ryan A. Palmer[3], Liam J. O'Reilly[1], Isaac V. Chenchiah[4], Daniel Robert[1]

[1]School of Biological Sciences, University of Bristol, Bristol, United Kingdom

[2]Department of Evolutionary Morphology, Museum für Naturkunde–Leibniz Institute for Evolution and Biodiversity Science, Berlin, Germany

[3]School of Engineering Mathematics and Technology, University of Bristol, Bristol, United Kingdom

[4]School of Mathematics, University of Bristol, Bristol, United Kingdom

*Corresponding author: Sam J. England

Email: sam.england@mfn.berlin

This PDF file includes:

Supporting text
Figures S1 to S3
Tables S1 to S2
SI References

Supporting Information Text

Supplementary results: wingbeat frequency measurements

In order to obtain a measure of the wingbeat frequency of the predators and mutualists with which treehoppers interact, ensuring stimulus relevancy, acoustic recordings were made of the predatory wasp *Mischocyttarus basimacula* (N = 2), and the mutualist bee species *Scaptotrigona subobscuripennis* (N = 75), and *Tetragonisca angustula* (N = 104). Predatory wasps were captured from their nest and immediately transferred to a mesh enclosure (≈ 30 × 30 × 30 cm). Their spontaneous flights within the enclosure were then recorded with a Samson Go Mic USB microphone (SAMSON AG, Frankfurt am Main, Germany). Mutualist bees were recorded acoustically by mounting a lavalier microphone at the entrance to their nests such that bees would naturally fly past it as they entered or exited the hive. All acoustic analyses were subsequently performed on the recordings in Audacity. The dominant frequency, assumed to be the fundamental frequency of the wingbeat, was extracted to a precision of 1 Hz from individual fly-bys using the plot spectrum tool, generating an FFT with Hann windowing and 4096 FFT lines. Prior to analysis, a high-pass filter was applied to wasp wingbeat recordings to remove excessive background noise, attenuating frequencies below 85 Hz with a roll-off of 24 dB per octave.

Analysis of acoustic recordings of flying predators and mutualists resulted in successful extraction of their wingbeat frequencies, with clear fundamental frequency peaks visible in the spectra, alongside multiple harmonics. These extractions yielded an average (mean ± SD) wingbeat frequency of 183 ± 3 Hz for the predatory wasp *Mischocyttarus basimacula* (N = 2), 139 ± 7 Hz for the stingless bee *Scaptotrigona subobscuripennis* (N = 75), and 188 ± 7 Hz for the stingless bee *Tetragonisca angustula* (N = 104). Example spectra of both a predatory and a mutualist species can be seen in Figure S3.

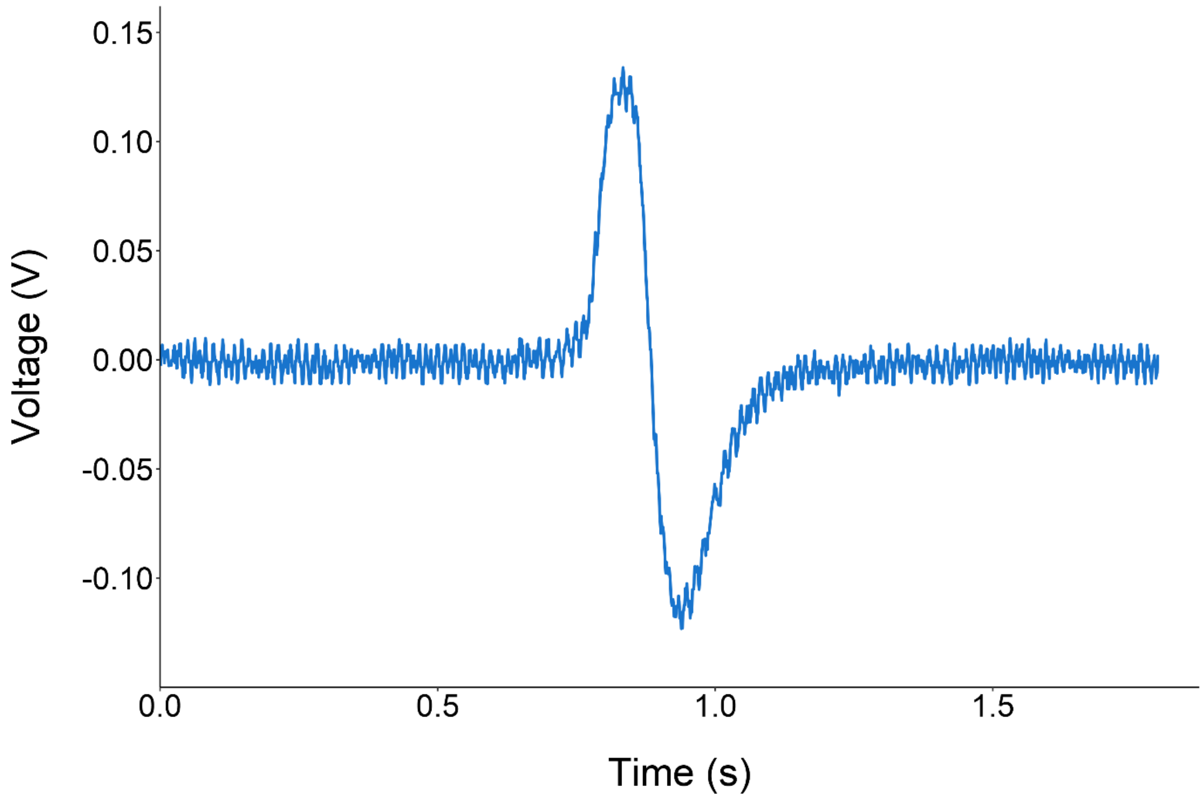

Figure S1: A typical example of the voltage generated by the picoammeter over time as a charged treehopper passes through the ring electrode system. An offset has been applied to bring the baseline to zero, and a notch filter between 49 and 51 Hz is applied to reduce 50 Hz noise from mains electricity in the United Kingdom. This particular example was an individual of the species *Polyglypta costata* carrying a net electrostatic charge of +1.85 pC.

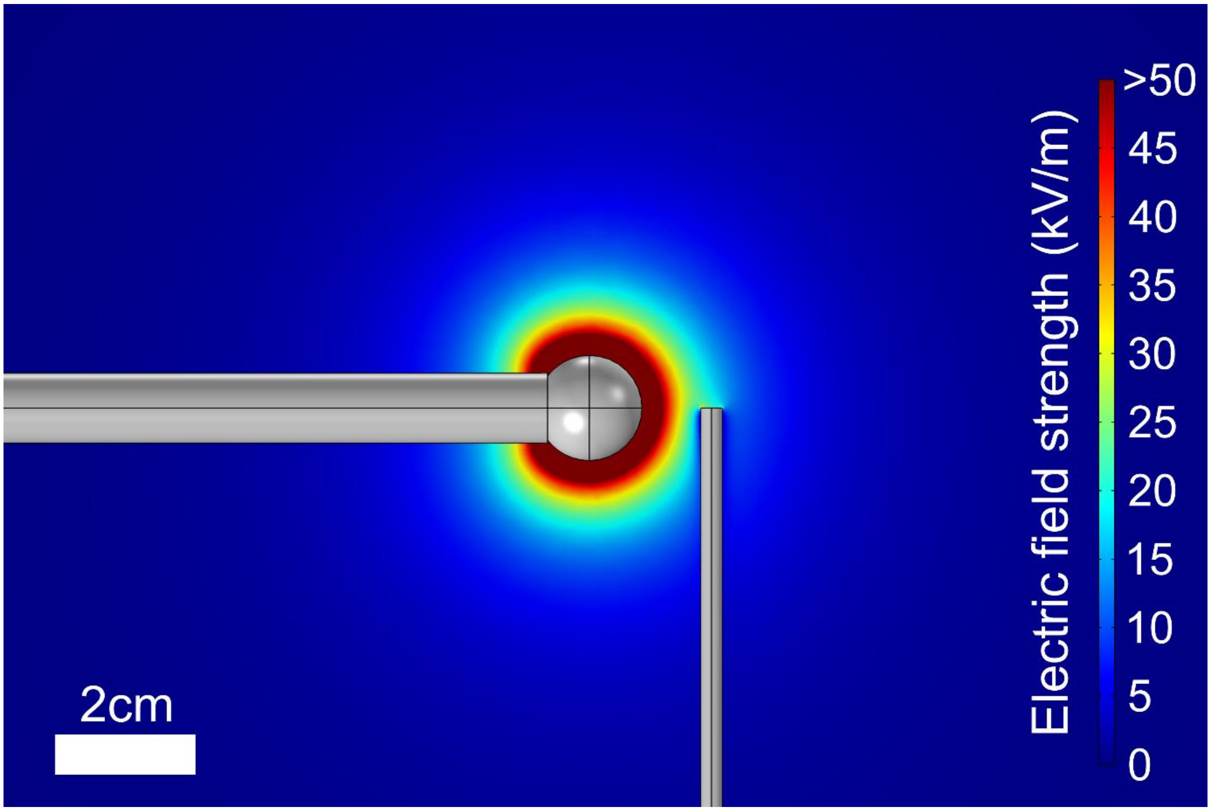

Figure S2: Three-dimensional finite element analysis of the experimental apparatus utilised for testing Poppea capricornis treehoppers for behavioural responses to electric fields. Colour gradient indicates electric field strength, with data truncated at 50 kVm^{-1} for clarity. Grey indicates model geometry.

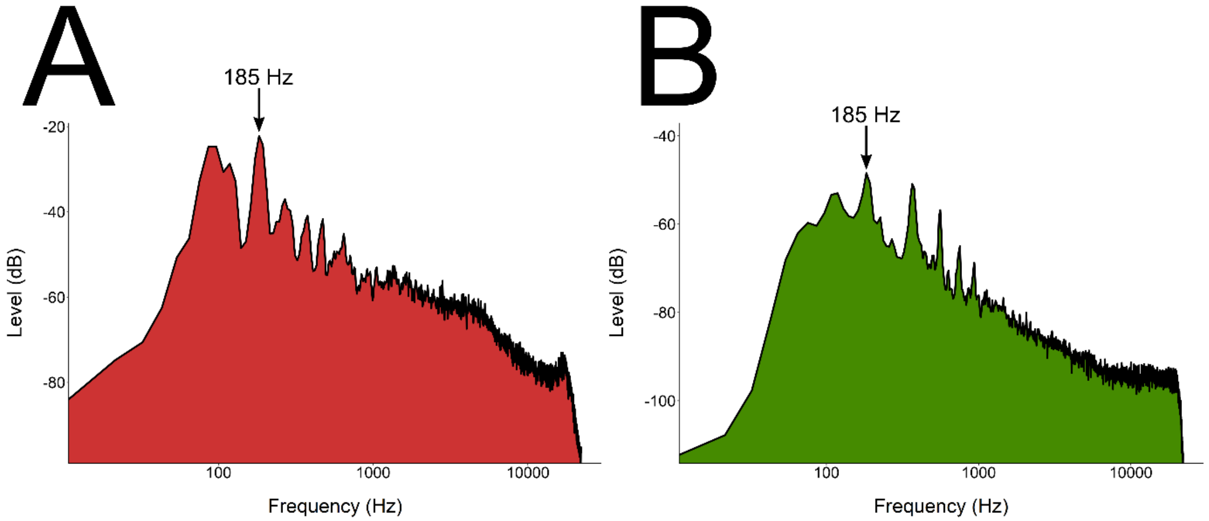

Figure S3: Example acoustic spectra of hymenopterans in flight. Spectra produced by Hann window FFTs with 4096 FFT lines. Arrows indicate dominant frequency, presumed to be the fundamental frequency of each specific individual's wingbeat. **(A)** The predatory wasp *Mischocyttarus basimacula*. **(B)** The mutualist stingless bee *Tetragonisca angustula*.

Table S1: The electrical conductivity, σ, and relative electric permittivity, ε_r, defined for each material included in the finite element analyses, alongside the sources of these values.

**Material**	**σ (S/m)**	**ε_r**	**Source(s)**
Air	1×10^{-14}	1	(1)
Plant tissue	N/A	35	(2, 3)
Insect tissue	N/A	80	Approximated to water
Wood	2.857×10^{-18}	2	COMSOL
Aluminium	3.774×10^{7}	1 (N/A)	COMSOL
Silicone	5.16×10^{-12}	4.5	(4)

Table S2: Inventory of the treehopper specimens subjected to morphological examination under light microscopy, along with their quantities, and sources. NHM = Natural History Museum, London, UK. RCC = Reginald B. Cocroft Collection, Missouri, USA. ZMH = Museum der Natur Hamburg – Zoologie, Hamburg, Germany.

Genus	Species	Sample size	Source
Aconophora	*marginata*	3	NHM
Antianthe	*expansa*	3	RCC
Bocydium	*amischoglobum*	2	ZMH
	globulare	4	NHM/RCC
	globuliferum	2	ZMH
	nigrofasciatum	1	ZMH
Cladonota	*apicalis*	4	NHM/RCC
Cyphonia	*clavata*	2	RCC
Funkhouseriana	*cerulea*	1	NHM
Gargara	*genistae*	3	NHM
Hemikyptha	*punctata*	3	NHM
Heteronotus	*spinosus*	3	NHM
	trinodosus	1	RCC
Membracis	*trimaculata*	3	NHM
Notocera	*tripodia*	3	NHM
Polyglypta	*costata*	3	NHM
Poppea	*capricornis*	2	RCC
Stylocentrus	*ancora*	3	NHM
Tomogonia	*vittatipennis*	1	RCC
Umbonia	*crassicornis*	5	NHM

SI References

1. K. A. Higazi, J. A. Chalmers, Measurements of atmospheric electrical conductivity near the ground. *Journal of Atmospheric and Terrestrial Physics* **28**, 327–330 (1966).
2. S. Dadshani, *et al.*, Non-invasive assessment of leaf water status using a dual-mode microwave resonator. *Plant Methods* **11**, 1–10 (2015).
3. F. T. Ulaby, R. P. Jedlicka, Microwave dielectric properties of plant materials. *IEEE Transactions on Geoscience and Remote Sensing* **GE-22**, 406–415 (1984).
4. H. T. Chiu, Y. L. Liu, C. W. Lin, Z. J. Shong, P. A. Tsai, Thermal conductivity and electrical conductivity of silicone rubber filled with aluminum nitride and aluminum powder. *Journal of Polymer Engineering* **33**, 545–549 (2013).
